# Perturbational Blowup Solutions to the Two-Component Dullin-Gottwald-Holm System

**DOI:** 10.1155/2016/5632798

**Published:** 2016-03-31

**Authors:** Ka Luen Cheung

**Affiliations:** Department of Mathematics and Information Technology, The Hong Kong Institute of Education, 10 Lo Ping Road, Tai Po, New Territories, Hong Kong

## Abstract

We construct a family of nonradially symmetric exact solutions for the two-component DGH system by the perturbational method. Depending on the parameters, the class of solutions includes both blowup type and global existence type.

## 1. Introduction

In this paper, we consider the following two-component Dullin-Gottwald-Holm (DGH) system:

(1) ρt+uρx=0,t>0,  x∈R,mt−Aux+umx+2uxm+γuxxx+ρρx=0,t>0,  x∈R

with

(2) $$\begin{array}{c} m=u-u_{xx}. \end{array}$$

Here, *u*(*t*, *x*):[0, *∞*) × *ℝ* → *ℝ* represents the horizontal velocity of the fluid and *ρ*(*t*, *x*):[0, *∞*) × *ℝ* → [0, *∞*) is related to the free surface elevation from equilibrium. *m* is the momentum density. Moreover, the constants *A* > 0 and *γ* ∈ *ℝ* are linear dispersion parameters.

In fluid dynamic, the investigation of shallow water wave models is an important subject and has attracted the attention of many researchers. One of the reasons is that the dynamics of tsunamis behave essentially as shallow water wave. More precisely, usually caused by earthquake in deep ocean, tsunamis have relatively small amplitude initially. Furthermore, the wavelength of tsunami is usually as huge as 200 kilometers, which is far greater than the depth of the ocean which is usually 2-3 kilometers. Thus, the behavior of tsunami is a large-scale version of shallow water wave.

Two-component DGH system (1) models wave-current interactions and was derived from the Euler equation with constant vorticity in shallow water moving over a linear shear flow. Its local well-posedness has been established in [1]. It is known that the system is completely integrable and can be written as a compatibility condition of two linear systems [1].

For *ρ*≢0 and *γ* = 0, system (1) becomes the two-component Camassa-Holm (CH) system [2] which describes water waves in the shallow water regime with nonzero constant vorticity. In particular, the CH equation (when *ρ* ≡ 0) enjoys two remarkable features, namely, the breaking wave phenomenon and the present of solutions in the form of peaked solitary waves or “peakons,” which make the CH system a better model for shallow water waves than KdV equation. Readers may refer to [3–7] for more information.

For *ρ* ≡ 0 and *m* = *u* − *α* ^2^ *u*_*xx*_, where *α* is a positive constant, system (1) becomes the DGH equation [8] which models unidirectional propagation of surface waves on shallow water. The DGH equation is completely integrable with a bi-Hamiltonian and a Lax pair. Moreover, its solutions include both the KdV solitons and the CH peakons as limiting cases [8]. For more details, readers may refer to [9–15].

This paper concerns the construction of exact solutions for system (1). In [16], the author constructed exact solutions for the two-component Camassa-Holm system by the perturbational method. We observe the similarities between the two-component Camassa-Holm system and system (1) and apply the perturbational method to system (1) to obtain some blowup solutions and solutions which globally exist. To the best of authors' knowledge, the construction of exact blowup solutions of the two-component DGH system in this paper first appears in the mathematics community. As main results, we have the following two theorems.

**Theorem 1 .**

For the two-component DGH system (1), there exists a family of solutions given by

(3) ρt,x=max⁡ρ2t,0−2b˙+bαa4/33t−Aa˙3ta3tx−3αa4/33tx2,0,ut,x=a˙3ta3tx+bt

with

(4) ρ2t,0=∫0te∫0s2a˙3r/a3rdr2bsb˙s+3bsa˙3s/a3sds+βe∫0t2a˙3r/a3rdr,

where *a*(3*t*) is a solution of the equation

(5) $$\begin{array}{c} a^{1/3}\left(\;3t\;\right)\ddot{a}\left(\;3t\;\right)=\alpha \end{array}$$

with initial data

(6) a0≕a0>0,a˙0≕a1

and *b*(*t*) is a solution of the following equation:

(7) b¨+6a˙3ta3tb˙+12αa4/33t+18αa˙3ta7/33t−6a˙23ta23t−18a˙33ta33tb=3Aαa4/33t.

Here, *α*, *β*, *a*_0_ > 0 and *a*_1_ are constants and can be arbitrarily chosen.

**Theorem 2 .**

(a) If one sets *α* < 0, then solutions (3)–(7) in Theorem 1 blow up in a finite time *T*.

(b) If one sets *α* = 0 and *a*_1_ < 0, then solutions (3)–(7) in Theorem 1 blow up in a finite time *T* = −*a*_0_/*a*_1_.

(c) If one sets *α* = 0 and *a*_1_ ≥ 0, then solutions (3)–(7) in Theorem 1 exist globally.

(d) If one sets *α* > 0, then solutions (3)–(7) in Theorem 1 exist globally.

## 2. Lemmas

It is well known that the solution to the Cauchy problem of any ODE exists locally and is unique provided that the given functions are smooth enough. Moreover, we have the following.

**Lemma 3 .**

If *p*(*t*), *q*(*t*), and *g*(*t*) are continuous on [*a*, *b*], then the differential equation

(8) $$\begin{array}{c} y^{\prime \prime }+p\left(\;t\;\right)y^{\prime }+q\left(\;t\;\right)y=g\left(\;t\;\right) \end{array}$$

with initial data

(9) yt0=y0,y′t0=y0′

has a unique solution defined for all *t* ∈ [*a*, *b*].

**Lemma 4 .**

For the differential equation

(10) $$\begin{array}{c} a^{1/3}\left(\;s\;\right)\ddot{a}\left(\;s\;\right)=\alpha \end{array}$$

with initial data

(11) a0≕a0>0,a˙0≕a1,

one has the following:

(a) If one sets *α* < 0, then there exists a finite time *T* such that
  (12) $$\begin{array}{c} \underset{s\to T^{-}}{lim}a\left(\;s\;\right)=0. \end{array}$$
(b) If one sets *α* = 0 and *a*_1_ < 0, then the solution *a*(*s*) blows up in the finite time *T* = −*a*_0_/*a*_1_.
(c) If one sets *α* = 0 and *a*_1_ ≥ 0, then the solution *a*(*s*) exists globally.
(d) If one sets *α* > 0, then the solution *a*(*s*) exists globally.

**Remark 5 .**

For a proof of Lemma 3, reader may refer to [17]; for a proof of Lemma 4, reader may refer to Lemma 3 of [18].

**Remark 6 .**

It is known in [1] that the solutions of the two-component DGH system blow up in finite time *T* > 0 if and only if

(13) $$\begin{array}{c} \underset{t\to T^{-}}{lim}\left\{\;\underset{x\in R}{\inf}u_{x}\left(\;t,x\;\right)\;\right\}=-\infty . \end{array}$$

This typical behavior is illustrated by solutions (3)–(7). More precisely, from (3)_2_, we have

(14) $$\begin{array}{c} u_{x}\left(\;t,x\;\right)=\frac{\dot{a}\left(3t\right)}{a\left(3t\right)}. \end{array}$$

From Lemma 4 (a), we know (13) happens since *a*(*t*) → 0^+^ and $\dot{a}(t)\to -\sqrt{a_{1}^{2}-3\alpha a_{0}^{2/3}}<0$ as *t* → *T* ^−^, for some finite *T* > 0.

## 3. Proofs of Theorems 1 and 2

The terms “perturbational solution” and “perturbation method” originate in [19]. The term “perturbational solution” is used to emphasise the existence of drifting term *b*(*t*) in the velocity of the form *u*(*t*, *x*) = *c*(*t*)*x* + *b*(*t*). The term “perturbation method” is used to emphasise that it is able to handle the solutions, where the velocity has a drifting term *b*(*t*). More precisely, the approach is explained as follows.

First, substitute the velocity form *u* = *c*(*t*)*x* + *b*(*t*) into the second equation of system (1). As *u* is linear in *x*, we see that the effect of some terms becomes irrelevant. One of the key properties of the first equation of system (1) is that one is able to express *ρ* ^2^(*t*, *x*) in terms of *ρ*(*t*, 0), *b*, *c*, *x*, and *x* ^2^. Another key property of system (1) is that one is able to transform the second equation into a system of *ρ* ^2^(*t*, *x*). Hence, one obtains a relation in *ρ* ^2^(*t*, 0), *b*, *c*, *x*, and *x* ^2^.

Second, set the coefficients of 1, *x*, and *x* ^2^, which are functions of *t*, to be zero. Then, one can obtain three ODEs. As the ODE induced by the term *x* ^2^ depends only on *c*, one can apply a backward analysis. More precisely, we use the Hubble transformation $c=\dot{a}/a$ to determine *c*. Then, the ODE induced by the term *x* depends only on *b* and *a* and is linear in *b*. Thus, *b* can be determined by classical theories in ODE. Finally, the ODE induced by the term 1 depends on *ρ* ^2^(*t*, 0), *b*, and *a* and is a first-order ODE in *ρ* ^2^(*t*, 0). Thus, a solution can be solved by the integral factor method. As *ρ* ^2^(*t*, *x*) can be expressed in terms of other determined solutions, a complete family of exact solutions is thus constructed. The detailed mathematics are implemented as follows.

First, we set

(15) $$\begin{array}{c} u\left(\;t,x\;\right)=c\left(\;t\;\right)x+b\left(\;t\;\right)=cx+b. \end{array}$$

Then, (1)_2_ becomes

(16) $$\begin{array}{c} -\rho \rho_{x}=\left[\;\dot{c}+3c^{2}\;\right]x+\left[\;\dot{b}+3bc-Ac\;\right]. \end{array}$$

It follows that

(17) $$\begin{array}{c} -\frac{1}{2}{\left(\;\rho^{2}\;\right)}_{x}=\left[\;\dot{c}+3c^{2}\;\right]x+\left[\;\dot{b}+3bc-Ac\;\right]. \end{array}$$

Integrating the above equation, one obtains

(18) ρ2t,x=ρ2t,0−c˙+3c2x2−2b˙+3bc−Acx.

On the other hand, from (1)_1_, one has

(19) ρt+uρx+uxρ=0,

(20) ρρt+uρρx+uxρ2=0,

(21) 12ρ2t+u12ρ2x+uxρ2=0.

Substituting (15) and (18) into (21) and grouping the coefficients of *x* ^2^, *x*, and 1, respectively, one gets

(22) $$\begin{array}{c} -\frac{1}{2}\left[\;\frac{d}{dt}\left(\;\dot{c}+3c^{2}\;\right)+4c\left(\;\dot{c}+3c^{2}\;\right)\;\right]x^{2} \end{array}$$

(23) $$\begin{array}{c} -\left[\;\ddot{b}+6\dot{b}c+4b\dot{c}+6bc\dot{c}+6bc^{2}-A\dot{c}-3Ac^{2}\;\right]x \end{array}$$

(24) $$\begin{array}{c} +\left[\;\frac{1}{2}\frac{d}{dt}\rho^{2}\left(\;t,0\;\right)+c\rho^{2}\left(\;t,0\;\right)-b\left(\;\dot{b}+3bc-Ac\;\right)\;\right]=0. \end{array}$$

We then set the coefficients to be zero and solve the ODEs step by step.

**Step 1 .**

From (22), we get

(25) $$\begin{array}{c} \frac{d}{dt}\left(\;\dot{c}+3c^{2}\;\right)+4c\left(\;\dot{c}+3c^{2}\;\right)=0. \end{array}$$

Letting

(26) $$\begin{array}{c} c\left(\;t\;\right)=\frac{\dot{a}\left(3t\right)}{a\left(3t\right)}, \end{array}$$

one has

(27) $$\begin{array}{c} \dot{c}+3c^{2}=3\frac{\ddot{a}\left(3t\right)}{a\left(3t\right)} \end{array}$$

and hence (25) becomes

(28) $$\begin{array}{c} 3\frac{\dddot{a}\left(3t\right)}{a\left(3t\right)}+\frac{\dot{a}\left(3t\right)\ddot{a}\left(3t\right)}{a^{2}\left(3t\right)}=0 \end{array}$$

or

(29) $$\begin{array}{c} 3a\left(\;3t\;\right)\dddot{a}\left(\;3t\;\right)+\dot{a}\left(\;3t\;\right)\ddot{a}\left(\;3t\;\right)=0. \end{array}$$

Multiplying the above equation by *a* ^−2/3^(3*t*) on both sides, one can reduce (29) to the following equation for *a*(3*t*) ≠ 0:

(30) $$\begin{array}{c} a^{1/3}\left(\;3t\;\right)\ddot{a}\left(\;3t\;\right)=\alpha \end{array}$$

with initial data

(31) a0≕a0>0,a˙0≕a1.

Here, *α* can be arbitrarily chosen.

**Step 2 .**

From (23), we get

(32) $$\begin{array}{c} \ddot{b}+f_{1}\left(\;t\;\right)\dot{b}+f_{2}\left(\;t\;\right)b=f_{3}\left(\;t\;\right), \end{array}$$

where

(33) f1t=6c=6a˙3ta3t,f2t=4c˙+6cc˙+6c2=12αa4/33t+18αa˙3ta7/33t−6a˙23ta23t−18a˙33ta33t,f3t=Ac˙+3Ac2=3Aαa4/33t.

Note that, by Lemma 3, the solution of (32) exists as far as *a*(3*t*) exists and is not zero.

**Step 3 .**

From (24), we obtain

(34) $$\begin{array}{c} F^{\prime }\left(\;t\;\right)+2cF\left(\;t\;\right)=G\left(\;t\;\right), \end{array}$$

where

(35) Ft≔ρ2t,0,Gt≔2bb˙+3bc−Ac.

The solutions of (34) are

(36) $$\begin{array}{c} F\left(\;t\;\right)=\frac{\int_{0}^{t}\mu \left(s\right)G\left(s\right)ds+\beta }{\mu \left(t\right)}, \end{array}$$

where

(37) $$\begin{array}{c} \mu \left(\;t\;\right)=e^{\int_{0}^{t}2c\left(r\right)dr} \end{array}$$

and *β* is an arbitrary constant.

Substituting (36) back into (18), we obtain solutions (3) and (4).

The proof of Theorem 1 is completed.

The proof of Theorem 2 follows directly from Lemma 4.

## 4. Conclusion

Exact solutions of differential equations play an important role in the proper understanding of qualitative features of many phenomena and processes in various areas of natural science. They graphically demonstrate and allow unraveling the mechanisms of many complex nonlinear phenomena such as spatial localization of transfer processes, multiplicity or absence steady states under various conditions, existence of peaking regimes, and many others. It is significant that many equations of physics, chemistry, and biology contain empirical parameters or empirical functions. Exact solutions allow researchers to design and run experiments, by creating appropriate natural conditions, to determine these parameters or functions.

In this paper, we constructed a family of exact solutions for two-component DGH system (1) by the perturbational method. The family of solutions depends on two classes of ODEs which their existence of solutions is analyzed. We also classified the parameters of the ODEs which lead to both finite time and global solutions of the original family of exact solutions. The typical behavior that the solutions of the two-component DGH system blow up in finite time if and only if the derivative of *u* with respect to the spatial variable becomes unbounded in finite time is illustrated by the exact solutions of Theorem 1.

We expect that the perturbational method for constructing exact solutions of nonlinear PDEs can be applied to more systems in the future.
